# For plantar taping, direction of elasticity matters

**DOI:** 10.1038/s41598-023-50169-2

**Published:** 2023-12-20

**Authors:** Dustin A. Bruening, Cody L. Messick, Davis C. Waid, Tanner D. Krupp, Jessica R. Stringer, Dylan J. Parry, Levi J. Berry

**Affiliations:** 1 Exercise Sciences Department, Brigham Young University, Provo, UT 84602 USA; 2 Mechanical Engineering Department, Brigham Young University, Provo, UT USA; 3 Finance Department, Brigham Young University, Provo, UT USA; 4 Canyon Foot and Ankle, Spanish Fork, Utah, USA

**Keywords:** Biomedical engineering, Bioenergetics, Translational research, Ligaments, Skeletal muscle

## Abstract

Plantar taping has been used in clinical settings as a short-term conservative treatment for plantar heel pain and related pathologies. The rise of at-home taping methods may offer patients more independence, but effectiveness has not been established. The purpose of this study was to evaluate the effects of plantar taping on foot mechanics during gait. We hypothesized that material compliance would drive mechanical effectiveness, with longitudinally inelastic tape reducing medial longitudinal arch (MLA) motion and anterior/posterior (A/P) plantar tissue spreading forces, and laterally inelastic tape reducing medial/lateral (M/L) tissue spreading. We also hypothesized that these effects would be influenced by foot structure. Fifteen healthy participants were tested in a randomized cross-over study design. Barefoot (BF) plus four taping methods were evaluated, including two inelastic tapes (Low-Dye, LD, and FasciaDerm, FD) along with longitudinally elastic kinesiology tape (KT) and a novel laterally elastic kinesiology tape (FAST, FS). Participants’ arch height and flexibility were measured followed by instrumented gait analysis with a multi-segment foot model. Ankle eversion and MLA drop/rise were calculated from rearfoot and forefoot reference frames, while plantar tissue spreading was calculated from shear stresses. ANOVAs with Holm pairwise tests evaluated tape effects while correlations connected arch structure and taping effectiveness (α = 0.05). The three longitudinally inelastic tapes (LD, FD, FS) reduced MLA drop by 11–15% compared with KT and BF. In late stance, these tapes also inhibited MLA rise (LD by 29%, FD and FS by 10–15%). FS and FD reduced A/P spreading forces, while FD reduced M/L spreading forces compared with all other conditions. Arch height had a moderately strong correlation (r = -0.67) with the difference in MLA drop between BF and FS. At-home plantar taping can affect the mechanical function of the foot, but tape elasticity direction matters. Longitudinally elastic kinesiology tape has little effect on mechanics, while inelastic tapes control MLA drop but also restrict MLA rise in late stance. Lateral elasticity does not limit tissue spreading and may increase comfort without sacrificing MLA control. At-home taping has the potential to broaden conservative treatment of plantar heel pain, flat foot deformity, and related pathologies, but additional studies are needed to connect mechanics with symptom relief.

## Introduction

Plantar heel pain (PHP) is a common pathology that impairs foot function and mobility and is often treated through a variety of conservative interventions. Also known as plantar fasciitis, PHP involves inflammation or degeneration of the plantar fascia which provides support to the foot's medial longitudinal arch (MLA). PHP affects approximately 10% of adults^1,2^, with two-thirds of these seeking clinical treatment^3^. Risk factors are thought to be primarily biomechanical^4^, including high activity levels (standing or athletics)^5–7^, obesity^8,9^, and potentially pes planus^10,11^. Symptoms of PHP typically include pain under the heel or in the arch of the foot, commonly occurring immediately after weight bearing or periods of extended inactivity. This pain can be both acute and chronic, at times lasting for several months. Treatment methods for PHP are primarily conservative, consisting of stretching and physical therapy, custom orthotics, plantar taping, steroid injections, and shock wave therapy^12^. Of these, taping methods may provide a practical treatment that can be used outside of the clinic; yet, additional research is needed to establish their effectiveness.

Traditionally, the Low-Dye (LD) taping method has been considered a gold standard in taping care, with several studies showing short-term pain reduction^13^. In LD taping, several strips of inelastic fabric tape are applied to the plantar surface of the foot to provide support. Tape is applied in an open-chain position with the 1st ray manually plantarflexed to shorten the plantar fascia, so that the tape will shield some of the strain when the MLA becomes loaded. In theory, this should reduce MLA deformation, although previous studies have focused measurements primarily on frontal plane rearfoot motion due to its potential coupling with the MLA^14,15^. Studies on static stance have found reduced peak rearfoot eversion^16^ as well as modified peak plantar pressures (although the locations of the pressure reductions are not consistent)^17^. Fewer effects have been noted during dynamic movements like gait, but a slight decrease in rearfoot eversion during early stance is likely^17^. The main limitation of LD taping is that, due to the complex application, it is typically only performed by a trained health care provider in a clinical setting. Thus, its use and compliance are often intermittent or acute only, potentially limiting its effectiveness on chronic PHP. The rise of simpler taping methods (i.e. single or double tape strips) that could potentially be applied at-home may offer patients more independence and control over their treatment, and could transform taping as an effective conservative treatment for PHP.

While promising, the effects of at-home taping methods have not been clearly established in the literature. One recent systematic review concluded that there was a modest short-term reduction in reported pain with kinesiology tape that was comparable to low-dye taping^18^. However, there is limited research on the mechanisms that might influence this pain reduction, particularly because kinesiology tape is typically applied with longitudinal elasticity, making it less likely to mechanically support the MLA. Two studies have evaluated the mechanical effects of these tapes, both evaluating only static stance in participants with either pes planus or excessively pronated feet. One showed no effect using elastic kinesiology tape^19^, while the other showed an increase in arch height using a more inelastic tape^20^. No group-level studies have evaluated the mechanical effects of taping on dynamic activities like gait.

The overall purpose of this study was to investigate the effects of various plantar taping methods on foot mechanics during walking gait. We performed this study on young healthy individuals using controlled taping methodology in order to minimize confounding influences, treating this as an important first step to understanding taping mechanisms prior to increasing ecological validity. We measured and compared foot mechanics, primarily MLA range of motion (RoM) and plantar tissue spreading^21^, while walking with different taping methods. We hypothesized that traditional inelastic tape (e.g. LD) would reduce MLA motion as well as anterior/posterior (A/P) tissue spreading compared to barefoot. We also expected that elastic kinesiology tape that is oriented longitudinally would reduce medial/lateral (M/L) tissue spreading but not MLA motion or A/P tissue spreading. Conversely, laterally oriented kinesiology tape would reduce MLA motion and A/P spreading but not M/L spreading. A secondary study purpose was to determine whether the magnitude of these effects are influenced by foot structure, hypothesizing that tape influence would be greater for flat or flexible feet. This secondary aim was motivated by the potentially higher incidence of PHP in flat feet^10,11^ as well as the additional potential application of plantar taping in flatfoot deformity independent of PHP^22,23^. An increased understanding of plantar taping mechanisms will ultimately influence their use in the treatment of foot pathology.

## Methods

### Design and participants

This was a randomized cross-over study design with each person testing all taping conditions during the same session. Fifteen young healthy participants (10 M/5F, age 23.1 ± 2.2 years, height 173.9 ± 8.5 cm, mass 75.3 ± 17.4 kg) were recruited from the local community through a snowball sampling method. Informed consent was obtained from all participants. All participants signed consent forms that were approved by the Brigham Young University institutional ethics board. Each participant was screened for foot pain and injuries that would affect normal gait.

### Types of taping

**Figure 1:**
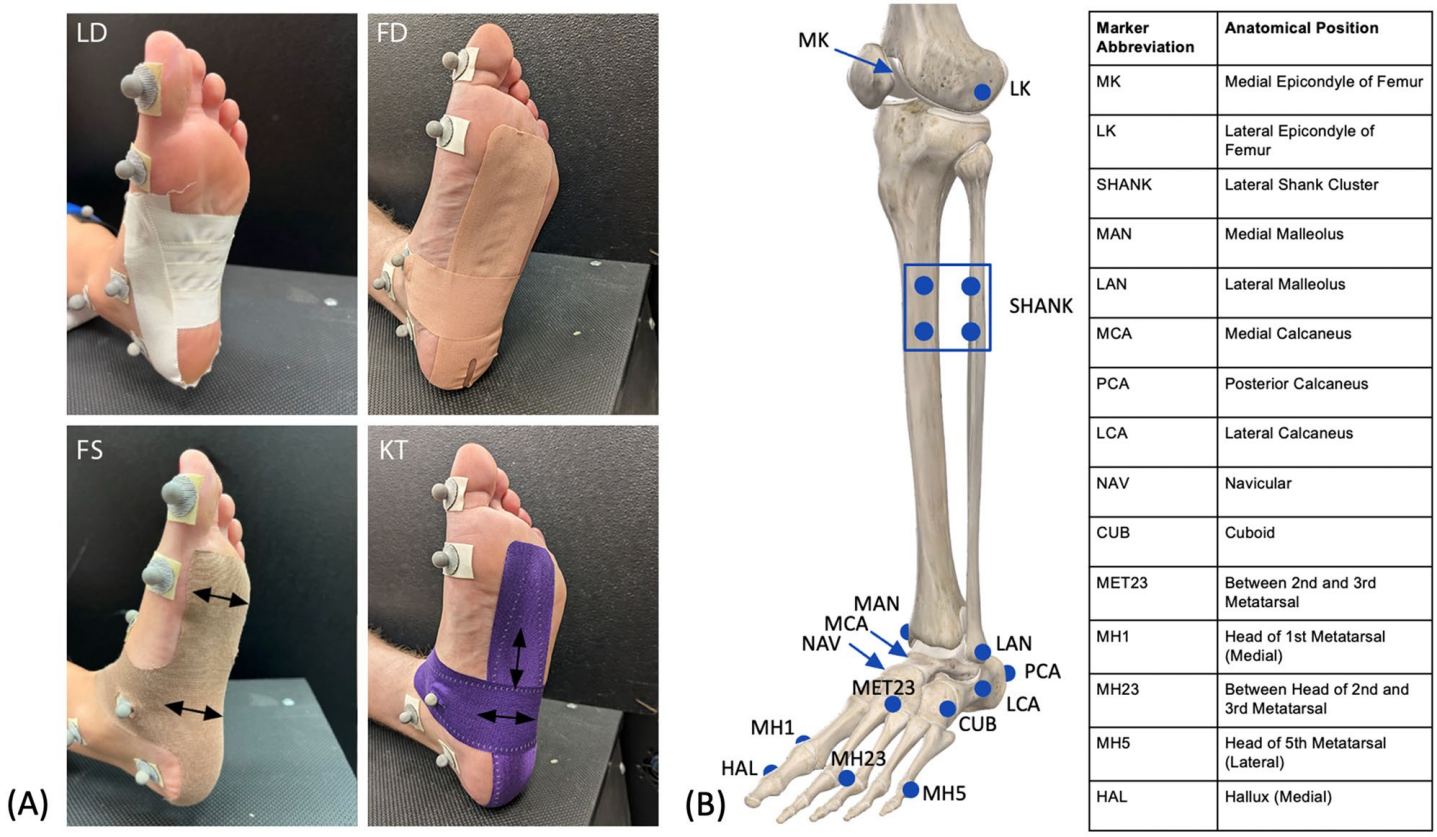
Taping methods and marker placement. (**A**) The four taping methods are shown: 1. Low Dye (LD), 2. Fasciaderm (FD), 3. FAST tape (FS), and 4. Kinesiology tape (KT). Black arrows denote the direction of material stretch for the two elastic kinesiology tape-based methods (FS and KT). (**B**) Markers were placed on the anatomical positions shown in the image and described on the chart. Actual markers are shown on the photographs in A. When the tape interfered with the marker locations (e.g. navicular and cuboid), a small hole was cut in the tape so that the marker remained visible.

Five different conditions were tested, consisting of four taping methods (Fig. 1) with barefoot as a baseline comparison:

1. LD: Low Dye taping method, applied using inelastic cloth tape (Wet-Pruf, Kendall International, Inc. Mansfield, MA, USA), with one strip circumferentially applied to the outer edges of the foot and 3–5 smaller strips M/L.
2. FD: Inelastic cloth tape applied as two pre-cut strips (Fasciaderm, Mueller Sports Medicine, Prairie du Sac, WI, USA), one applied A/P, the other M/L.
3. FS: Custom cut kinesiology tape (initial commercial name is FAST Tape^24^), applied as one pre-cut cross-shaped strip that is inelastic longitudinally (E/P), but elastic laterally (M/L).
4. KT: Rectangular kinesiology tape with lengthwise elasticity (KT Tape, American Fork, UT, USA), applied as two strips, one A/P the other M/L.
5. BF: Barefoot, no tape applied.

Taping methods 2–4 followed manufacturer recommendations, while the LD method was chosen to capture common criteria for low dye taping.

### Protocol

**Figure 2:**
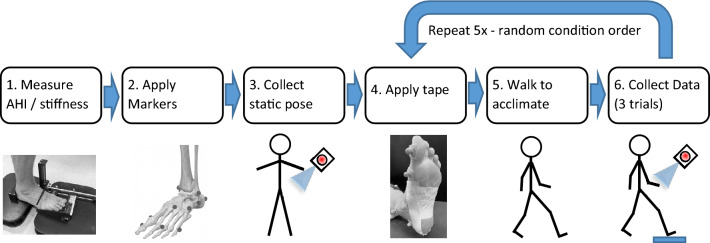
Data collection flowchart.

Prior to applying tape, seated and standing MLA heights were measured using the Arch Height Index Measurement System (AHIMS, JAK Tool & Model, NJ, USA). Next, 18 retroreflective markers were adhered to the left lower limb according to a custom multi-segment foot model (Fig. 1). A barefoot static pose was collected to calibrate the model. The first randomized taping condition was then applied to the left foot. The tape was applied without removing markers. Tape was applied by research team members with clinical training and supervision from a licensed podiatrist. For consistency, the same researchers applied the tape for all conditions. Prior to collection, the subject walked around the lab for one minute to acclimate to the tape and allow it to settle. The subject then walked at a controlled speed (1.3 m/s) across a raised 5.5 m long walkway containing an embedded shear stress sensor (footSTEPS, ISSI, inc. Dayton, OH USA)^25^. Speed was monitored by timing lights (Brower, inc. Draper UT USA) and repeated if not within target range (± 0.065 m/s) or if the left foot was not within the sensor boundaries. Starting placement was adjusted by the researchers to avoid purposeful targeting of the sensor. Shear data was collected at 50 Hz while marker trajectories were captured at 100 Hz using a 12-camera motion analysis system (Qualysis, Gottberg, Sweden). After three acceptable trials, the tape was removed (without removing the markers) and the steps above were repeated for each of the five randomized conditions (Fig. 2).

### Data analysis

Two MLA structure metrics were calculated from the AHI measurements for analysis: standing arch height index^26^ and sit-to-stand arch flexibility^27^. Linear correlations were run among these two metrics and early stance MLA RoM difference (α = 0.05), choosing for the latter the RoM metric/taping condition post-hoc as the one having the greatest effect (see “Results”).

The multi-segment foot model was implemented using Visual 3D software (C-motion, Germantown, MD USA). The model was based on prior validation work^28,29^ but simplified for the current purpose. It consisted of shank, rearfoot, and forefoot segments, separated by ankle and midtarsal joints. Segment orientations were calibrated on the barefoot static pose, aligning each reference frame’s vertical axis with the laboratory (thus removing any structural differences in orientation to focus only on ranges of motion). Marker trajectories were low-pass filtered (6 Hz cutoff). A common Euler rotation sequence (1-Sagittal, 2-Frontal, 3-Transverse) was used to calculate angular positions for both joints; however, analysis focused only on the frontal plane ankle angle and the sagittal plane midtarsal angle. An additional measure of midtarsal angular displacement was also calculated—a novel signed helical angle (SHA) algorithm. Briefly, this measures helical, or axis-angle, displacements between the rearfoot and forefoot segments, thus capturing the tri-planar MLA motion in a single measure. SHA algorithm details are found in Bassett et al.^30^. For each kinematic measurement, RoM metrics were extracted during stance. Ankle eversion RoM was defined as the frontal plane ankle RoM from initial contact to maximum eversion. Midtarsal drop was defined as the sagittal plane midtarsal RoM from initial contact to lowest position, while midtarsal rise was the RoM from lowest to subsequent highest position. Midtarsal pronation and supination were defined in the same manner as midtarsal drop and rise, but using the SHA values.

**Figure 3:**
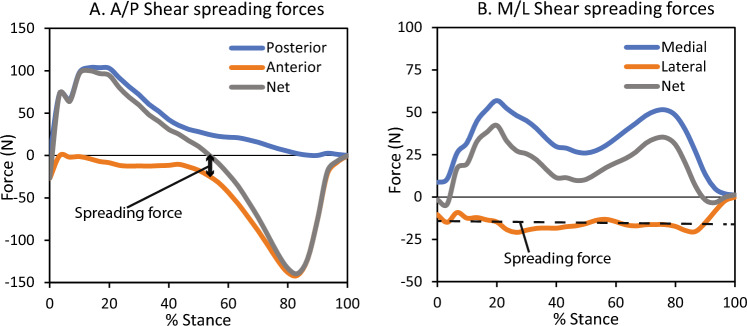
Plantar tissue spreading analysis methodology. A/P and M/L shear forces from a representative participant are shown, plotted against time normalized stance phase. Directional shear forces are calculated from stresses multiplied by sensel area and summed by direction (i.e. posterior separate from anterior, medial separate from lateral). Net forces are also shown for reference. The A/P spreading force was extracted at midstance, where the net force crossed zero—this is the location of maximum spreading (i.e. opposing) shear forces. Spreading was quantified by the anterior or posterior (equal to each other) force at this point. The M/L spreading force was extracted as the mean lateral force (which opposes the primary medial force) across stance.

Plantar tissue spreading metrics were extracted from the measured A/P and M/L shear stresses (Fig. 3)^21,31^. A/P spreading was defined as the maximum opposing shear force at midstance (when net force transitioned from posterior to anterior). M/L spreading was defined as the mean opposing shear force throughout stance.

The effect of taping condition on each of the seven chosen metrics (5 kinematic, 2 kinetic) was evaluated statistically using repeated measures ANOVAs (α = 0.05). For each ANOVA, Mauchly’s test for sphericity was tested and corrected for if necessary. The Benjamini–Hochberg procedure, with a false discovery rate of 0.1, was used to account for the multiple main effects comparisons. When significant main effects were found, Holm post-hoc tests were run to determine pairwise comparisons. Pairwise effect sizes were also calculated using Cohen’s d, but for brevity only the largest effect size compared to barefoot was presented. In addition, mean kinematic waveforms were plotted for visual descriptive comparisons.

### Ethics approval and consent to participate

Human subject testing approval was obtained from the Brigham Young University ethics committee (protocol # IRB2022-039). Written informed consent was obtained from all participants and all methods were performed in accordance with the relevant guidelines and regulations.

## Results

The standing AHI of all participants was 0.32 ± 0.03 while the sit-to-stand arch flexibility was 16.6 ± 4.5 mm/kN.

**Figure 4:**
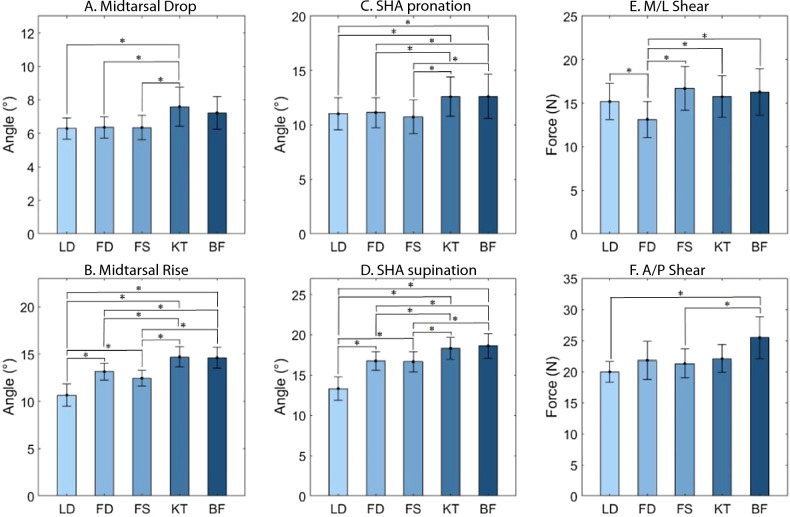
Pairwise comparisons across conditions for the six metrics showing main effect significance. Bar plots show means with standard deviation bands. Significant pairwise comparisons are denoted by * symbol above brackets connecting the associated pair.

There was no significant main effect for ankle eversion RoM, but there were for the other two early stance metrics (Table 1).Midtarsal drop and SHA pronation showed somewhat similar pairwise results (Fig. 4). For SHA pronation (Fig. 4C), the three longitudinally inelastic tapes (LD, FD, FS) showed similar RoMs that were less than the longitudinally elastic KT tape and the BF control. For midtarsal drop (Fig. 4A), the same three tapes were also less than KT, but the comparisons with BF did not quite reach significance. In both metrics, effect sizes for these tapes compared to BF were medium (e.g. 0.55 and 0.57).

**Table 1:** Metric comparisons across conditions (mean ± st dev).

Metric	LD	FD	FS	KT	BF	*p*-value	Cohen’s d
Ankle eversion (°)	5.8 ± 2.7	5.2 ± 1.9	5.6 ± 2.2	5.5 ± 2.3	5.9 ± 1.9	0.175	0.32 (FD)
Midtarsal drop (°)	6.3 ± 1.3	6.4 ± 1.3	6.3 ± 1.5	7.6 ± 2.3	7.2 ± 1.9	0.001*	0.55 (LD)
SHA pronation (°)	11.0 ± 3.0	11.1 ± 2.8	10.7 ± 3.1	12.6 ± 3.6	12.6 ± 4.0	< 0.001	0.57 (FS)
Midtarsal rise (°)	10.7 ± 2.3	13.1 ± 1.8	12.4 ± 1.7	14.8 ± 2.1	14.6 ± 2.2	< 0.001*	1.95 (LD)
SHA supination (°)	13.3 ± 2.9	16.7 ± 2.3	16.7 ± 2.5	18.3 ± 2.7	18.6 ± 3.1	< 0.001*	1.26 (LD)
A/P spreading (N)	20.0 ± 3.4	21.8 ± 6.2	21.3 ± 4.6	22.1 ± 4.5	25.5 ± 6.7	0.002*	1.05 (LD)
M/L spreading (N)	15.2 ± 4.1	13.2 ± 4.1	16.7 ± 5.0	15.7 ± 4.8	16.3 ± 5.3	< 0.001*	0.67 (FD)

Condition acronyms are: *LD* low dye, *FD* fasciaderm, *FS* FAST tape, *KT* kinesiology tape,* BF* Barefoot.

Main effect p-values are displayed, with * denoting statistical significance (α = 0.05, with additional Benjamini–Hochberg procedure). A Cohen’s d pairwise effect size is also presented, representing the largest pairwise effect size with respect to barefoot (with the chosen condition following in parentheses).

Both late stance metrics also showed significant main effects (Table 1). Midtarsal rise (Fig. 4B) and SHA supination (Fig. 4D) were practically identical, with all pairwise comparisons significant except for BF vs. KT and FS vs. FD. In order, LD showed the least motion, followed by FS/FD, and BF/KT having the greatest. LD demonstrated large effect sizes compared to BF (1.95 and 1.26), with medium to large effect sizes for FS and FD (0.70–1.07).

**Figure 5:**
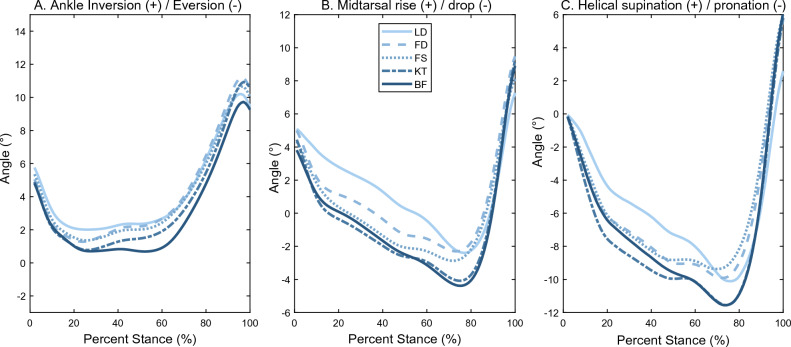
Mean (across participants) angular waveforms for each condition: (**A**) Frontal plane ankle motion, (**B**) Sagittal plane midtarsal motion, and (**C**) Signed helical angle (SHA) midtarsal motion.

Visually, all kinematic waveforms (Fig. 5) followed the same general trends, with three visual groupings (BF/KT, FD/FS, and LD). While the chosen RoM metrics captured the main features, LD also displayed a markedly delayed deformation (i.e. slope) in early stance compared with the other conditions.

Significant main effects were also found for both spreading metrics (Table 1). In the A/P direction, shear spreading forces were reduced in FS and LD compared to BF (Fig. 4F), with large effect sizes (e.g. 1.05). In the M/L direction, shear spreading was less in FD compared with all other conditions (Fig. 4E), with a medium effect size compared to BF (0.67).

The greatest taping effect was found between BF and FS in SHA pronation, thus the correlations were run on this difference. AHI had a significant (p = 0.007) and moderately strong (r = − 0.67) correlation with pronation difference, while AH stiffness did not quite reach significance (p = 0.052, r = 0.51). AHI and AH stiffness were also not correlated with each other (p = 0.348).

## Discussion

The primary purpose of this study was to investigate the effects of various plantar taping methods on midfoot joint and plantar tissue mechanics. We focused on taping methods that could be available for at-home application and compared these tapes with Low Dye, a traditional clinically applied inelastic taping method, as well as a barefoot control. Our hypotheses were mostly upheld, with longitudinally inelastic tapes (LD, FS, and FD) generally reducing arch motion and A/P shear spreading forces and laterally inelastic tapes (LD, FD, and KT) reducing M/L shear spreading forces. Our secondary purpose, investigating the influence of foot posture, was partially upheld, with lower MLA heights showing a greater tape influence.

Our primary measurements revolved around the MLA deformation or drop that occurs from initial contact through mid-stance, which has a direct relationship with plantar fascia strain. During these phases, the rearfoot everts about the subtalar joint and the MLA drops in a somewhat coupled manner^14,15^. We captured the former with our ankle eversion RoM and the latter with two separate but similar measures (midtarsal drop and SHA pronation). While ankle eversion did not quite reach significance, the differences in mean waveforms were visually consistent with both MLA variables, i.e. potential differences were in the same directions, just not quite significant (Fig. 5). The strongest results were seen in the novel SHA pronation metric, likely because it captures some of the non-sagittal plane motion that may also be a part of the tri-planar MLA motion^14^. The combined results also suggest that with additional participants we might have seen significance in all three variables. Both midtarsal drop and SHA pronation should have a direct relationship with plantar fascia strain^15,32,33^. This fascia runs the length of the foot, crossing the midfoot joints as well as the metatarsophalangeal joints; however, since these latter joints are typically in a neutral position in mid-stance^28^, any reduction in MLA drop due to taping should also result in a reduction in fascia strain^32,33^. This could in theory relieve some of the effects associated with PHP, for example. As hypothesized, we found that the longitudinally inelastic tapes LD, FD and FS all reduced MLA drop (and consequently plantar fascia strain) by similar amounts (11–15%) when compared to BF. In contrast, the longitudinally elastic KT had no effect on the MLA. These results are consistent with the limited prior work on kinesiology tape^19^ and inelastic tape^20^ in standing. Without a mechanical explanation, the previously observed reduction in pain from kinesiology tape^18^ is most likely due to something other than MLA control, such as changes in mechanoreceptor function^21^ or blood/lymph flow^34^. In addition, it should be noted that while LD reductions were similar to FD and FS, the initial waveform slope was visually quite different (Fig. 5B,C), with an apparent delay in deformation. It is possible that LD affected mechanics in early stance in a way that was not fully captured in our RoM analysis; the implications of this difference should be explored in future clinical studies.

The effect of FS tape on MLA drop (using SHA pronation) was significantly correlated with standing AHI, suggesting a potential relationship between taping effects and foot structure. The correlation between pronation difference and AHI was negative, with lower AHIs (i.e. flatter MLAs) associated with greater taping effects. This may simply be due to a greater potential for manipulating the position of the MLA during tape application in lower arched feet. AH stiffness was close to, but not quite, significant, and may be worthy of future investigation (the correlation was positive, suggesting potentially greater flexibility associated with greater pronation effects). However, the lack of a correlation between AHI and AH stiffness also suggests that these two measurements are capturing slightly different aspects of foot structure. While we only tested healthy participants, the fairly strong relationship with AHI is encouraging given the potential for pathologies/injuries in flatfoot deformity^35,36^. AHI and AH stiffness values for our participant sample are also comparable to previous normative studies—our mean AHI (0.322) was similar to Williams et al.^37^ (0.328) but slightly lower than Butler et al.^26^ (0.340), while our median AH stiffness values (16.2) were slightly higher than Zifchock et al.^27^ (14.75, no mean reported).

While reduced MLA RoM in early and mid-stance is likely beneficial in reducing plantar fascia strain, longitudinally inelastic tape also affects MLA rise in late stance, which could have a mildly detrimental effect on gait energetics. In late stance, MLA rise contributes to propulsive power generation^38^, and a small reduction in MLA power is likely compensated for elsewhere (e.g. more proximal joints). All three longitudinally inelastic tapes inhibited MLA rise compared to BF; however, LD had a more than twofold greater effect (27–29%) than FD and FS (10–15%). The reduction in MLA rise could be caused by factors other than mechanical restriction, including discomfort or neural inhibition, and is also likely accompanied by reductions in toe extension^29^. In addition, if some of this power generation is due to release of stored energy from MLA deformation earlier in stance^39^, there may be no way to prevent it while still controlling for MLA deformation.

In addition to MLA motion, we also evaluated the effects of taping on other plantar tissues through our directional shear force metrics. While the relationship between these metrics and taping effectiveness is not as straightforward, they provide some initial insights into the effects of taping on both joint mechanics and plantar skin deformation. In the A/P direction, the greatest spreading forces occur in mid-stance, when the arch is loaded and the forefoot and rearfoot generate opposing actions (much like a loaded truss), thus part of this spreading likely overlaps with the joint RoM results. Indeed, our A/P spreading results closely matched our MLA RoM results, although FD did not quite reach significance compared with barefoot. In the M/L direction, the laterally inelastic FD decreased spreading; however, LD and KT did not. For LD, this is likely due to the tape location, which leaves open the areas under the heel and forefoot where the greatest spreading occurs^31^. For KT, the second tape strip reverses the fiber direction to be elastic in the M/L direction, thus its effect here was limited. The M/L spreading results add entirely new information, but the implications are not fully clear. We hypothesize that M/L spreading is at least partially related to comfort, and allowing for M/L spreading could be beneficial proprioceptively, if not also mechanically. Additional research is needed to confirm this.

Study limitations primarily revolved around ecological validity and clinical significance, as our measurements focused on joint and plantar tissue mechanics in healthy participants in a lab based setting. We stopped short of estimating plantar fascia strain itself—this could be done using a simple truss model^40^ or a more complex musculoskeletal model—however, we did not see an interpretation advantage of strain over joint RoM. We focused on mechanical differences through p-values, % differences, and effect sizes, but assigning clinical meaning to any of these measures would likely require connections with pain or other symptoms in affected populations. We performed this study on healthy young adults in order to control for gait and structural abnormalities, avoid increasing risk in symptomatic patients, and focus solely on taping method mechanisms. While the mechanical effects are informative and promising, there is a need to study taping implementation in symptomatic patients (e.g. PHP and flatfoot deformity), older adults, and athletes. Related, we also standardized our taping application procedures, and acknowledge that additional variability will likely be present when tape is applied in at-home settings. We chose common methodology and documented our approach, but there are many ways to apply some of the tapes (particularly LD) that could yield slightly different results. In addition, all of the tapes likely settle, stretch, and/or break down over time, and while we included a short break-in period, we did not evaluate long term use. Follow up studies are needed to verify time-varying taping effectiveness when applied by patients with foot pathologies in at-home settings. Finally, we relied on skin mounted markers to track foot segment motion. We employed a within-subject design to eliminate marker placement differences and focused analysis on RoM to minimize the effects of any potential skin repositioning due to tape application (i.e. skin stretch under a marker affecting the initial pose). However, we cannot rule out the possibility of an interaction between the tape and skin/marker motion itself that could have introduced additional errors.

## Conclusions

Overall, our study suggests that plantar tape elasticity direction matters when it comes to mechanical effects. The multi-strip, bi-directionally rigid LD taping method was effective at limiting MLA motion; however, it typically requires clinical application, limiting its use and compliance. The commercially available FD tape had similar results to LD and initially appears to be an effective alternative that could be applied at-home. FS tape was introduced as a novel tape in this study, with encouraging results. While matching FD in MLA variables, it may have an advantage in allowing for more M/L tissue spreading, potentially increasing comfort and proprioception. On the other hand, KT, with its opposite elasticity directions, resulted in no mechanical differences compared with barefoot. Thus, any effects on pain are likely proprioceptive rather than mechanical. These results can inform clinical implementation now but should also be followed up with studies on clinical populations and at-home implementation.

## Data Availability

The datasets used and/or analyzed during the current study are available from the corresponding author on reasonable request.
